# Gender comparison of clinical, histopathological, therapeutic and outcome factors in 185,967 colon cancer patients

**DOI:** 10.1007/s00423-019-01850-6

**Published:** 2020-01-31

**Authors:** Rosa Schmuck, Michael Gerken, Eva-Maria Teegen, Isabell Krebs, Monika Klinkhammer-Schalke, Felix Aigner, Johann Pratschke, Beate Rau, Stefan Benz

**Affiliations:** 1 Chirurgische Klinik, Charité-Universitätsmedizin Berlin, Campus Mitte I Campus Virchow-Klinikum, Chariteplatz 1, 10117 Berlin, Germany; 2 Tumorzentrum Regensburg–Institut für Qualitätssicherung und Versorgungsforschung der Universität Regensburg, Am BioPark, 93053 Regensburg, Germany; 3 Arbeitsgemeinschaft Deutscher Tumorzentren e.V, Kuno-Fischer-Strasse 8, 14057 Berlin, Germany; 4 Viszeralonkologisches Zentrum Klinikum Böblingen Sindelfingen, Arthur-Gruber-Straße 70, 71032 Böblingen, Germany

**Keywords:** Sex differences, Gender, Colon cancer

## Abstract

**Introduction:**

Colorectal carcinomas represent the third most common cause of cancer-related deaths in Germany. Although the incidence is significantly higher in men compared with women and gender is a well-established crucial factor for outcome in other diseases, detailed gender comparisons for colon cancer are lacking.

**Methods:**

This retrospective population-based cohort study included all patients diagnosed with colon cancer in Germany between 2000 and 2016 who were included in the common dataset of colorectal cancer patients from the quality conference of the German Cancer Society. We compared clinical, histopathological, and therapeutic characteristics as well as overall and recurrence-free survival.

**Results:**

A total of 185,967 patients were included in the study, of which 85,685 were female (46.1%) and 100,282 were male (53.9%). The proportion of women diagnosed with colon cancer decreased from 2000 to 2016 (f: 26.6 to 40.1%; m: 24.9 to 41.9%; *p* < 0.001), and the proportion of very old patients was especially high in women (f: 27.3%; m: 15.6%; *p* < 0.001). The localization in women was more right-sided (f: 45.0%, m: 36.7%; *p* < 0.001), and women had a higher tumor grading and a higher UICC stage (especially stage III nodal-positive) at diagnosis of primary colon cancer (UICC III: f: 22.7%, m: 21.0%; *p* < 0.001). We could detect a significantly better overall (hazard ratio: 0.853, lower 95%: 0.841, upper 95%: 0.864; *p* < 0.001) and recurrence-free survival (hazard ratio: 0.857, lower 95%: 0.845, upper 95%: 0.868; *p* < 0.001) in women compared with men, even though women received chemotherapy less frequently compared with men (f: 26.1%, m: 28.1%; *p* < 0.001).

**Conclusion:**

We could detect several variables that differed significantly between men and women regarding clinical, histopathological, therapeutic, and outcome factors. We believe that it is crucial to consider gender as a key factor in the diagnosis and treatment of colon cancer. Sex-specific diagnostic tools could lead to an earlier diagnosis of colon cancer in women, and ways to increase the rate of chemotherapy in women should be evaluated. Furthermore, we recommend stratifying randomized trials by gender.

**Electronic supplementary material:**

The online version of this article (10.1007/s00423-019-01850-6) contains supplementary material, which is available to authorized users.

## Introduction

Colorectal cancer is one of the leading causes of cancer-related death worldwide [1]. Germany shows a particularly high incidence, with 58,900 projected new cases in 2018 (81.6 in100.000 for men and 62.6 in100 [2]). With the implementation of nationwide screening coloscopies in 2005, a steady decline in incidence and colon cancer-related mortality could be achieved [3]. However, most studies available do not address some very relevant patient characteristics. For instance, most clinical studies on the effect of chemotherapeutic regimens have been conducted with an upper age limit of 65 years. As more than half of the patients diagnosed with colon cancer are over 70 years of age and the very few studies analyzing this age group imply a strong effect of advanced age on therapy response, the results seem less convincing [4]. Another factor influencing survival is the gender of patients. A meta-analysis of 13 retrospective cohort studies and one randomized controlled trial demonstrated that the sex of a patient was the single significant predictor of the relative advantage of survival [5]. There have been attempts to explain this sex-dependent difference in survival. However, the reasons leading to this effect remain unclear. One possible influencing factor with therapeutic potential is the hormone balance [6]. Furthermore, sex-dependent differences in the administration of adjuvant chemotherapy have been described [7]. Strikingly, women have a higher risk of developing right-sided colon cancer than men, which is associated with a more aggressive form of neoplasia [1].

However, gender is often neglected in the experimental setup of preclinical and clinical studies and in current treatment algorithms. In addition, animal models are mainly carried out with male animals [8]. This imbalance is also translated into clinical trials: several analyses of inclusion rates of study participants in clinical cancer research show a tremendous underrepresentation of women: even in non-sex-specific cancer types, the rate of female participants was only 38.8% [9]. In a study published in YAMA, men were also more likely than women to enroll in colorectal cancer trials (enrollment fractions: 2.1% vs 1.6%, *p* < 0.001) [10]. This disparity can also be found in non-cancer-specific clinical trials [11]. Taking into account that gender is a factor that strongly influences diagnosis, treatment, and survival in cancer, this underrepresentation of women represents a strong bias, and it has to be assumed that the validity of the results in those studies is considerably impaired.

In the present study, we aim to provide a gender-focused analysis of national population-based data on colon carcinoma to take stock and possibly draw conclusions from the current status quo.

## Patients and methods

### Patients and database

This retrospective population-based cohort study is based on a national dataset of colorectal cancer patients from the quality conference of the German Cancer Society (Deutsche Krebs Gesellschaft DKG). This dataset combines data from 30 clinical registries, covering approximately 28% of the German population, gathered by the German Tumor Centres Work Group ADT (Arbeitsgemeinschaft Deutscher Tumorzentren e.V.) and the German Cancer Associations GEKID (Gesellschaft der epidemiologischen Krebsregister in Deutschland e.V.). All patients diagnosed with colon cancer (ICD-10 C18 according to the international classification of diseases) between January 1, 2000 and December 31, 2016 were included in the pooled analysis [12], no patients were excluded from analysis. In a separate subgroup, only patients with adenocarcinoma, without previous and simultaneous second tumor and with UICC stage I-III were analyzed. In this group, all patients underwent surgical resection. The dataset includes demographic data of patients and information on diagnosis, histology, TNM classification, (neo-) adjuvant and surgical therapy, and patient follow-up. Life status and recurrences of the patients were ascertained using clinical reports; additionally, life status was completed using death certificates from the local public health departments and information from the registration offices of the patients’ respective resident districts.

### Statistics

Quantitative and qualitative variables were expressed as the median (range) and frequency (percentage) as well as mean and standard deviation. Comparisons between groups were analyzed with the chi-squared or Fisher’s exact test for categorical variables and Student’s *t* test or the Mann–Whitney *U* test for continuous variables. The normal distribution of data was tested with the Kolmogorov-Smirnov normality test. The effect of categorical variables on the gender distribution was additionally estimated by means of multivariable binary logistic regression analysis, which renders adjusted odds ratios (OR) for the “chance” of a higher or lower proportion of women vs men in certain categories compared with a defined reference category.

Overall survival was calculated from the date of diagnosis to the date of death or last follow-up using the Kaplan–Meier method. To estimate recurrence-free survival, locoregional relapses and subsequent distant metastases were considered as additional events. A *p* value from the log-rank test of 0.05 or less was considered to be significant.

In the multivariable analyses, overall and recurrence-free survival were adjusted for year of diagnosis, age at diagnosis, tumor localization, second tumor, histological type, grading, and UICC stage. Patients with adenocarcinoma only, without previous and simultaneous second tumor and UICC stage I-III were analyzed as a separate subgroup (ACO). In the ACO group, alle patients underwent surgical resection, only 499 patients (0.4%) received neoadjuvant treatment. Here, 86.196 patients (74.0%) underwent partial colon resection, 5.1% a total or extended resection, 6.3% had resection with inclusion of rectum, and 14.6% had radical surgery without specification. Within this group, subgroups having different UICC stage I–III, age at diagnosis, and tumor localization were examined more closely for gender differences in recurrence-free survival (RFS). Therefore, the adjustment was extended to therapy variables residual tumor status, number of lymph nodes resected, and adjuvant chemotherapy. Furthermore, survival analysis was performed in a subgroup restricted to patients with R0-resection in stage UICC III who survived a postoperative interval of 90 days, when considering the effect of adjuvant chemotherapy.

All significance tests were two-sided with a significance level of 0.05; the results are displayed as *p* values or 95% confidence intervals (CI). For statistical analysis, SPSS (version 25; IBM Corp., Armonk, New York, USA) was used.

## Results

### Demographic, clinical and histopathological characteristics according to sex

A total of 85,685 female (46.1%; f) and 100,282 male (53.9%; m) patients were included in the study with a total study population of 185,967. A total of 62,566 patients (33.6%) were included in the ACO group.

Analyzing patients’ demographic, clinical, and histopathological characteristics according to sex, we found women to be significantly older than men when diagnosed with colon cancer (more than 80 years old: 27.3% f vs. 15.6% m, mean age 71.7 years f vs. 69.4 m, median age 73.6 vs 71.7, *p* < 0.001). However, the proportion of women decreased when comparing the time periods 2000–2004 and 2010–2016 (*p* < 0.001, Table 1).

**Table 1:** Patient demographic, clinical and histopathological characteristics according to sex in the overall cohort (*n* = 185,967). All differences in relative distributions are highly significant with *p* < 0.001 according to Pearson’s chi-square test

		Sex					
		Male		Female		Total	
		N	%	N	%	N	%
Year of diagnosis	2000–2004	24,947	24.9	22,831	26.6	47,778	25.7
	2005–2009	33,348	33.3	28,519	33.3	61,867	33.3
	2010–2016	41,987	41.9	34,335	40.1	76,322	41.0
Age at diagnosis	0–49	5261	5.2	4941	5.8	10,202	5.5
	50–59	12,795	12.8	9231	10.8	22,026	11.8
	60–69	29,663	29.6	18,779	21.9	48,442	26.0
	70–79	36,882	36.8	29,356	34.3	66,238	35.6
	80+	15,681	15.6	23,378	27.3	39,059	21.0
Tumor localization	Right colon	36,812	36.7	38,588	45.0	75,400	40.5
	Transverse colon	8419	8.4	7324	8.5	15,743	8.5
	Left colon	49,181	49.0	34,918	40.8	84,099	45.2
	Other locations	5870	5.9	4855	5.7	10,725	5.8
Tumor localization ICDO-3	Cecum	13,020	13.0	15,287	17.8	28,307	15.2
	Appendix	1725	1.7	2137	2.5	3862	2.1
	Right colon	16,151	16.1	16,614	19.4	32,765	17.6
	Right flexure	5916	5.9	4550	5.3	10,466	5.6
	Transverse colon	8419	8.4	7324	8.5	15,743	8.5
	Left flexure	3936	3.9	2766	3.2	6702	3.6
	Left colon	6065	6.0	4045	4.7	10,110	5.4
	Colon sigmoideum	39,180	39.1	28,107	32.8	67,287	36.2
	Overlapping loc.	1051	1.0	789	0.9	1840	1.0
	Other locations	4819	4.8	4066	4.7	8885	4.8
Second tumor (previous, synchronous)	No	97,846	97.6	84,151	98.2	181,997	97.9
	Yes	2436	2.4	1534	1.8	3970	2.1
Histological type	Adenocarcinoma	95,727	95.5	81,201	94.8	176,928	95.1
	Neuroendocr. Ca	1636	1.6	1824	2.1	3460	1.9
	Other carcinoma	2728	2.7	2470	2.9	5198	2.8
	Other tumor lesions	191	0.2	190	0.2	381	0.2
Grading	G1	6488	6.5	5184	6.1	11,672	6.3
	G2	65,544	65.4	52,578	61.4	118,122	63.5
	G3/4	21,029	21.0	21,666	25.3	42,695	23.0
	Unspecified	7221	7.2	6257	7.3	13,478	7.2
UICC stage	I	19,932	19.9	15,353	17.9	35,285	19.0
	II	25,381	25.3	22,234	25.9	47,615	25.6
	III	21,052	21.0	19,445	22.7	40,497	21.8
	IV	22,938	22.9	18,956	22.1	41,894	22.5
	X	10,979	10.9	9697	11.3	20,676	11.1
	Total	100,282	100.0	85,685	100.0	185,967	100.0

Women had more right-sided carcinomas than men (45.0% f vs. 36.7% m; *p* < 0.001); however, the most frequent location of tumors was the colon sigmoideum in both sexes. Remarkably, women had more poorly differentiated tumors (G3/4) than men and presented with a higher UICC stage (especially stage III nodal-positive, Supplementary Table 1; *p* < 0.001) at diagnosis of primary colon cancer. When comparing tumor entities, women were diagnosed more often with other tumors than adenocarcinomas, especially neuroendocrine tumors (2.1% f vs. 1.6% m; *p* < 0.001). The results are summarized in Table 1. All differences in relative distributions listed in the results part are highly significant with *p* < 0.001 according to Pearson’s chi-square test. The significance of all differences is confirmed by means of multivariable binary logistic regression (Supplementary Table 2).

When ACO patients were analyzed exclusively, all the described differences (age, tumor localization, distribution of histological types, grading, UICC stage) could be confirmed (*p* < 0.001; Supplementary Table 3).

### Patient treatment characteristics according to sex

For patient treatment characteristics, we analyzed ACO patients only (UICC stages I–III, adenocarcinoma only).

Regarding the number of removed lymph nodes, more lymph nodes were removed in female patients (≥ 24 lymph nodes: 25.2% f vs. 23.3% m; *p* < 0.001). However, this difference was only found in left-sided colon cancer when analyzing resected lymph nodes according to the location of the tumor and resection (right side: *p* = 0.296; left side: *p* < 0.001). Women showed a higher lymph node ratio (*p* < 0.001; number of positive lymph nodes/number of removed lymph nodes).

The rate of positive resection margin (R1) was more frequent in women; however, it was low overall (1.8% f vs. 1.6% m; *p* < 0.001). Remarkably, fewer women received chemotherapy after resection (26.1% f vs. 28.1% m; *p* < 0.001; overall study cohort, Table 2). The rates of adjuvant chemotherapy according to age group and patient sex with R0-resected nodal-positive colon carcinoma UICC stage III confirmed these findings. This observation could partly be explained by the fact that women were older when diagnosed with colon cancer: in the age group over 80 years, women received adjuvant chemotherapy less frequently (19.4%) compared with men (25.8%, *p* < 0.001). In the age groups under 80, the rates were comparable but constantly declining with age in both sexes (Supplementary Table 4). All analysis of chemotherapy refers to adjuvant treatment.

**Table 2:** Patient treatment characteristics according to sex in the ACO subgroup (UICC stages I–III, *n* = 116,528). All differences in relative distributions are significant according to Pearson’s chi-square test

		Sex					
		Male		Female		Total	
		N	%	N	%	N	%
Removed lymph nodes	LK >0– < 12	6625	10.6	5176	9.6	11,801	10.1
	LK > =12– < 24	33,392	53.4	28,819	53.4	62,211	53.4
	LK > = 24	14,560	23.3	13,615	25.2	28,175	24.2
	No information	7989	12.8	6352	11.8	14,341	12.3
Residual tumor stage	R0	55,221	88.3	47,504	88.0	102,725	88.2
	R1/2	1005	1.6	949	1.8	1954	1.7
	No information	6340	10.1	5509	10.2	11,849	10.2
Lymph node ratio	0/ns	44,796	71.6	37,450	69.4	82,246	70.6
	< 0.100	7071	11.3	6710	12.4	13,781	11.8
	0.100–0.199	4474	7.2	4077	7.6	8551	7.3
	0.200–0.399	3791	6.1	3436	6.4	7227	6.2
	0.400+	2434	3.9	2289	4.2	4723	4.1
Chemotherapy	Yes	17,557	28.1	14,076	26.1	31,633	27.1
	No	45,009	71.9	39,886	73.9	84,895	72.9
Mortality postoperative (30 days)	No	60,675	97.0	52,483	97.3	113,158	97.1
	Yes	1891	3.0	1479	2.7	3370	2.9
Mortality postoperative (90 days)	No	59,442	95.0	51,468	95.4	110,910	95.2
	Yes	3124	5.0	2494	4.6	5618	4.8
	Total	62,566	100.0	53,962	100.0	116,528	100.0

To address the question of the distribution of age and tumor grading in right-sided colon cancer in women, we further analyzed this subgroup. Women showed a higher grading of the tumor in all UICC stages (*p* < 0.001). Women were older in all tumor grades, and they were shown to not be younger in higher tumor grading groups (grading 3 or 4; *p* < 0.001).

### Survival

The mean follow-up for survival analysis was 7.8 years (median 7.7). Regarding postoperative mortality, women showed both a lower 30-day and 90-day mortality in the ACO group (*p* < 0.001; Table 2).

**Fig. 1:**
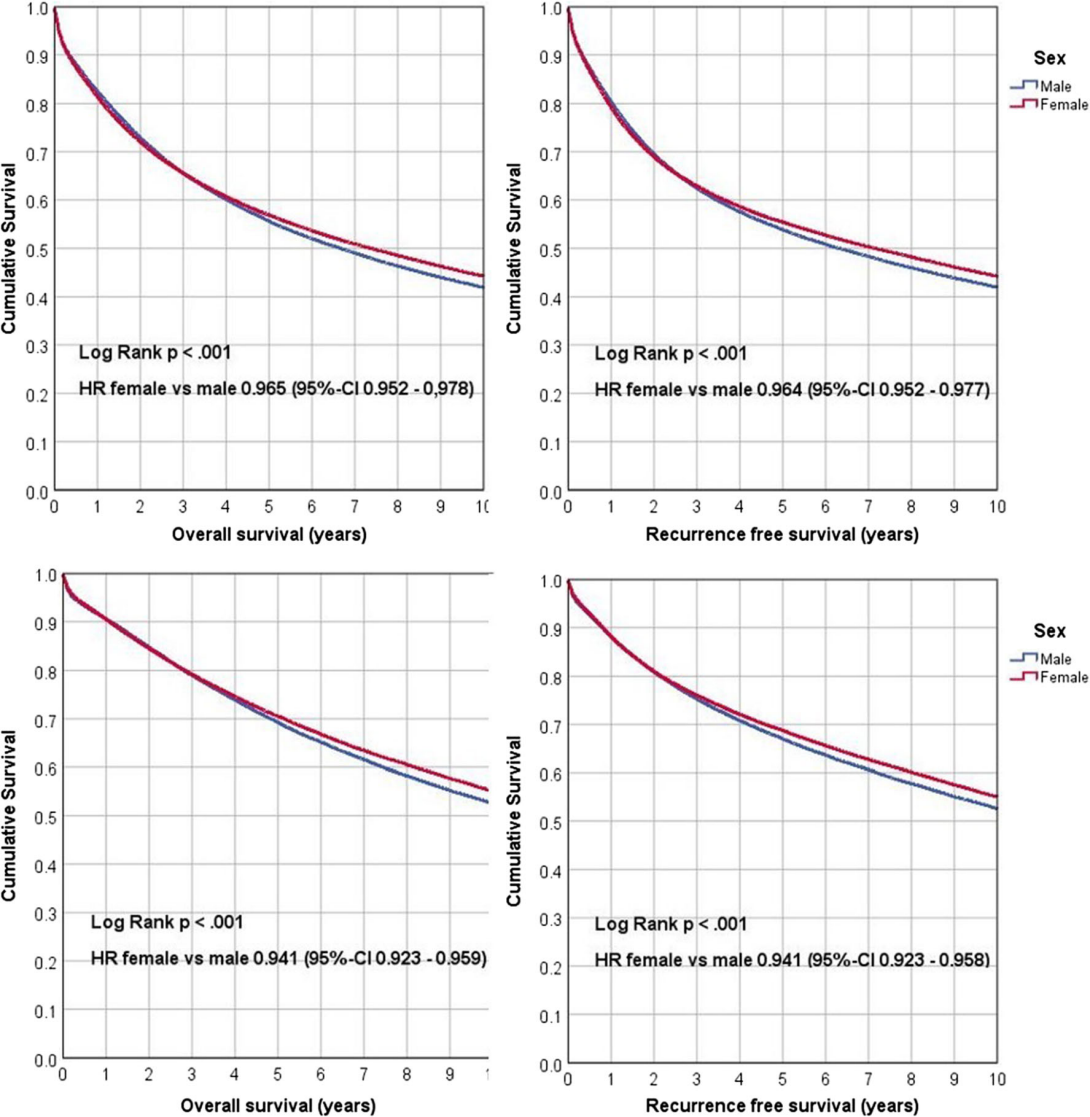
Kaplan-Meier survival plots for overall survival and recurrence-free survival of female and male patients: top UICC stages I–IV (*n* = 185,967), bottom ACO subgroup (UICC stages I–III, *n* = 116,528)

Women showed a slightly higher overall and recurrence-free survival rate compared with men in the univariate regression analysis. After adjustment for risk factors, better survival in women is even more prominent (*p* < 0.001). This survival benefit was also observed independently of the localization of the tumor (*p* < 0.001; left sided and right sided). This was confirmed both in the overall study cohort and in the ACO group in multivariate analysis but was more pronounced in the ACO group (*p* < 0.001; Table 4, Fig. 1). Here, the HR for OAS in women vs men was 0.795 (95% CI 0.780–0.811, *p* < 0.001), and for RFS, the HR was 0.802 (95% CI 0.787–0.818, *p* < 0.001).

The significant advantage for women in OAS and RFS persists in all subgroups for UICC stage I–III, age at diagnosis, and tumor localization, as shown for RFS in Table 5 (*p* < 0.001). The survival advantage of women compared with men is more pronounced in patients with UICC stage I compared with stages II and III (*p* < 0.001). Except for the group of patients who were younger than 50, the survival advantage of women as opposed to men is more pronounced in younger patients. No differences in survival advantage were observed with respect to tumor localization.

A significant benefit of adjuvant chemotherapy after R0 resection in nodal-positive patients can be stated for OAS and RFS in both males and females and the combined group (*p* < 0.001; Table 3). The benefit for women is slightly smaller for OAS (HR 0.760, 95% CI 0.716–0.805; *p* < 0.001) compared with men (HR 0.721, 95% CI 0.684–0.761; *p* < 0.001). An equal difference can be observed when RFS is examined (females: HR 0.820, 95% CI 0.774–0.869; males: HR 0.782, 95% CI 0.742–0.825; *p* < 0.001).

**Table 3:** Hazard ratios from univariable and multivariable Cox regression for overall survival and recurrence-free survival for chemotherapy vs no chemotherapy in patients with R0-resected nodal-positive colon carcinoma UICC stage III (*n* = 40,497). Multivariable survival analyses were performed for both sexes combined, separately for men and women, and adjusted for year of diagnosis, age at diagnosis, tumor localization, grading, and lymph nodes resected. Patients who died within 90 days after diagnosis were excluded

Group	Outcome	Risk Adjustment	*p*	Hazard ratio	Lower 95%	Upper 95%
Male and female	Overall survival	No, univariable	< 0.001	0.532	0.514	0.552
		Yes, multivariable*	< 0.001	0.746	0.717	0.776
	Recurrence-free survival	No, univariable	< 0.001	0.591	0.571	0.612
		Yes, multivariable	< 0.001	0.806	0.775	0.838
Male	Overall survival ACO	No, univariable	< 0.001	0.564	0.537	0.593
		Yes, multivariable*	< 0.001	0.721	0.684	0.761
	Recurrence-free survival ACO	No, univariable	< 0.001	0.627	0.597	0.659
		Yes, multivariable	< 0.001	0.782	0.742	0.825
Female	Overall survival ACO	No, univariable	< 0.001	0.494	0.470	0.520
		Yes, multivariable*	< 0.001	0.760	0.716	0.805
	Recurrence-free survival ACO	No, univariable	< 0.001	0.550	0.523	0.578
		Yes, multivariable	< 0.001	0.820	0.774	0.869

**Table 4:** Hazard ratios from univariable and multivariable Cox regression for overall survival and recurrence-free survival of female versus male patients with a mean follow-up of 7.8 years (median 7.7) in both the overall study cohort and ACO group. Multivariable survival analyses were adjusted for year of diagnosis, age at diagnosis, tumor localization, second tumor, histological type, grading, and UICC stage

Outcome	Risk Adjustment	*p*	Hazard ratio	Lower 95%	Upper 95%
Overall survival	No, univariable	< 0.001	0.965	0.952	0.978
	Yes, multivariable*	< 0.001	0.853	0.841	0.864
Recurrence-free survival	No, univariable	< 0.001	0.964	0.952	0.977
	Yes, multivariable	< 0.001	0.857	0.845	0.868
Overall survival ACO	No, univariable	< 0.001	0.941	0.923	0.959
	Yes, multivariable*	< 0.001	0.795	0.780	0.811
Recurrence-free survival ACO	No, univariable	< 0.001	0.941	0.923	0.958
	Yes, multivariable	< 0.001	0.802	0.787	0.818

**Table 5:** Hazard ratios from multivariable Cox regression for recurrence-free survival of female patients versus male patients in subgroups of UICC stage, age at diagnosis and tumor localization (adjusted for year of diagnosis, age at diagnosis, tumor localization, second tumor, histologic type, grading, UICC stage, removed lymph nodes, residual tumor stage, and chemotherapy), ACO group (*n* = 116,528)

Variable, factor	Category	*p*	Hazard ratio	Lower 95%	Upper 95%
UICC stage	I	< 0.001	0.688	0.658	0.719
	II	< 0.001	0.805	0.779	0.832
	III	< 0.001	0.832	0.805	0.859
Age at diagnosis	0–49	0.016	0.843	0.734	0.968
	50–59	< 0.001	0.685	0.631	0.745
	60–69	< 0.001	0.713	0.680	0.747
	70–79	< 0.001	0.790	0.764	0.816
	80+	< 0.001	0.858	0.827	0.891
Tumor localization	Colon right side	< 0.001	0.788	0.764	0.814
	Colon transversum	< 0.001	0.776	0.723	0.832
	Colon left side	< 0.001	0.793	0.769	0.818
	Colon others/overlap	< 0.001	0.818	0.745	0.898

## Discussion

This study represents the first gender-focused analysis of national population-based data on colon carcinoma in Germany. It reveals several differences between men and women with potential relevance for screening, diagnosis, and treatment of colon cancer. Women were significantly older than men when diagnosed with colon cancer and presented with more advanced disease. This might be caused by the fact that the acceptance of screening colonoscopies is especially lower in women older than 75 years compared with their male peers, as shown in a survey of statutory insured in Germany from 2003 to 2011 [13]. The effectiveness of this tool is highly accepted and led to a decrease in the incidence of approximately 14% for both sexes [3]. Another possible factor causing this effect is the higher rate of incomplete colonoscopies in women [1], [14]. This might also be caused by the fact that the standard coloscopy devices are often not suitable for women who tend to have a longer colon transversum and a smaller bowel diameter [15]. In clinical reality, thinner colonoscopy devices facilitate a complete colonoscopy until the ileocecal junction in women [16]. This is of particular importance given that right-sided carcinomas are more frequent in women, not only in our study cohort. The recent adjustment of the guidelines and coverage of screening colonoscopies now starting with 50 years for men instead of 55 years only bring additional benefit for men. Women would need screening colonoscopies later than the usually recommended 75 years, as 27.3% of women in our cohort were older than 80 years when diagnosed with colon cancer. However, studies suggest that the risk of colonoscopy-associated complications exceeds the benefit of screening, although studies do not distinguish between the sexes [17].

Interestingly, the administration of chemotherapy was lower in women than in men in our cohort both in the overall cohort and in patients with R0-resected nodal-positive colon carcinoma UICC stage III. This observation is mainly based on the lower rate of women above 80 years receiving chemotherapy compared with that of their male peers. In some other age groups, we could even detect an opposite trend. The overall lower rate of administration of adjuvant chemotherapy has been described for other regions; however, it has not yet been described in Germany [18]. Whether this is mainly caused by the older age of women at the time of diagnosis which has previously been discussed, our results support these findings, as the lower rate of adjuvant chemotherapy was particularly apparent in the age group over 80 years. However, it must be considered that analyses thereof are strongly dependent on local conditions (e.g., environmental, socioeconomic), and therefore, cross-regional comparisons have to be interpreted with caution.

Another influencing factor could be the higher rate of side effects in women: even though women are known to have a higher plasma volume and lean body mass, a reduced hepatic clearance, differences in the activity of cytochrome P450 enzyme, and metabolize drugs at an unequal rate to men, most dosage recommendations for chemotherapy are not gender specific [19]. This might lead to the greater side effects due to relative overdosing and reduce the rate of successful chemotherapy.

As described previously, female sex was associated with a higher number of removed lymph nodes in our cohort. The usually higher lymph node yield in right-side hemicolectomies compared with left-side hemicolectomies, and the finding that more women suffered from right-sided colon cancer [20], [21], however, could not explain this observation because this difference was only present in left-sided cancer.

The groundbreaking CALGB/SWOG 80405 study revealed a considerable survival benefit of 14 months for left-sided colon cancer compared with right-sided cancer [22]. In other studies, tumor location was confirmed as an independent prognostic factor, with some showing clearly distinct mutational patterns [23], [24]. As supported by our data, women suffer more frequently from right-sided colon cancer [25], [26]. The reason for this sex-specific laterality remains unclear. In our cohort, women neither did show younger age when suffering from right-sided colon cancer nor did younger patients show higher tumor grades. This could be interpreted as an indirect sign that hereditary syndromes such as Lynch syndrome, which are associated with high-grade dysplasia at a young age, preferably in the right colon, might not be the reason for the observed laterality of tumors in women. However, right-sided colon cancer tends to cause more unspecific symptoms and manifests later than left-sided cancer. This might be an additional cause for the higher tumor stages in women in our cohort.

Taking all of this into account, it is quite surprising that women in our cohort show a slight but significantly better overall survival than men. A meta-analysis by Yang et al. confirmed a longer overall survival for women when comparing nine studies [5]. Additionally, an analysis from Majek et al. confirmed these findings in a German cohort [27]. Considering the higher tumor grade, older age, lower rate of administration of chemotherapy, and higher number of right-sided cancers, other factors have to be responsible for this effect.

A possible explanation might be the protective effect of female hormones against colorectal cancer as described before [27], [28]. This lower risk is even more prominent in colon cancer patients under hormone substitution when compared with rectal cancer patients [28].

Two main underlying mechanisms might be the loss of estrogen inactivation and the estradiol activation in colon cancer [29], [30]. However, the WHI study (a randomized, double-blind, placebo-controlled trial involving 10,739 postmenopausal women) could not prove a protective effect of estrogen substitution on the incidence of colorectal cancer or deaths from or after colorectal cancer [31].

In the present study, we only observed a survival benefit in women older than 50 years. Therefore, hormone substitution in postmenopausal women has to be considered a strong influencing factor. Unfortunately, data on female hormone substitution in our cohort are missing. The results of three population-based surveys, analyzing data from 4.503 women aged 45–74 years in Germany between 1997 and 2003, showed a prevalence of hormone substitution of 17.0%. A lower use of hormone substitution was observed in women older than 65 years, and most women started hormone substitution between 44 and 49 years [32]. In our cohort, we observed the highest survival benefit in the age group between 50 and 59 years, an age where a high rate of hormone substitution can be suspected. However, a possible effect of the described lower use of hormone substitution in women older than 65 is not depicted in our data, as we can also see a significant survival benefit of those women compared with their male peers.

A lower postoperative mortality seems to not be the reason for the survival benefit because the effect is also stable after adjustment for 30- and 90-day mortality.

This underlies the importance of further investigating the role of gender in colon cancer not only based on analysis of clinical data but also focusing on the underlying mechanisms. Experimental setups including female animals in preclinical research as well as study designs including and clearly distinguishing both sexes in clinical studies are crucial steps towards this goal.

Several limitations of the study have to be addressed. Even though the results presented here are based on a fairly large patient number and include a long-time follow-up, registry based data do have limitations per se. Due to the large patient cohort, comparisons lead to statistical significant differences even if the clinical relevance might be limited. It has to be stated that the differences in the proportion of patients for some variables is very small. Conclusions of the differences obtained should therefore be drawn carefully. Some information (e.g., on the operation technique) was incomplete for the majority of patients so no analysis could be performed. In contrary to single center datasets, subsequent completion of data is not possible.

## Electronic supplementary material

ESM 1 (DOCX 38 kb)
