# Supplementary material for: Gender comparison of clinical, histopathological, therapeutic and outcome factors in 185,967 colon cancer patients
Source: Langenbecks Arch Surg. 2020 Jan 31;405(1):71–80. doi: 10.1007/s00423-019-01850-6 (PMC7036075; doi:10.1007/s00423-019-01850-6)
Supplement: Supplementary file 1 — (DOCX 38 kb) [file 423_2019_1850_MOESM1_ESM.docx]

| Variable, factor | | | | Sex | | | | | |
| --- | --- | --- | --- | --- | --- | --- | --- | --- | --- |
|  |  |  |  | Male | | Female | | Total | |
|  |  |  |  | Count | Layer Column N % | Count | Layer Column N % | Count | Layer Column N % |
| UICC Stage | I | Grading | G1 | 3131 | 3.1% | 2347 | 2.7% | 5478 | 2.9% |
|  |  |  | G2 | 14,090 | 14.1% | 10,725 | 12.5% | 24,815 | 13.3% |
|  |  |  | G3/4 | 1703 | 1.7% | 1589 | 1.9% | 3292 | 1.8% |
|  |  |  | Unspecified | 1008 | 1.0% | 692 | 0.8% | 1700 | 0.9% |
|  | II | Grading | G1 | 1195 | 1.2% | 1025 | 1.2% | 2220 | 1.2% |
|  |  |  | G2 | 18,663 | 18.6% | 15,283 | 17.8% | 33,946 | 18.3% |
|  |  |  | G3/4 | 4521 | 4.5% | 4983 | 5.8% | 9504 | 5.1% |
|  |  |  | Unspecified | 1002 | 1.0% | 943 | 1.1% | 1945 | 1.0% |
|  | III | Grading | G1 | 596 | 0.6% | 544 | 0.6% | 1140 | 0.6% |
|  |  |  | G2 | 13,764 | 13.7% | 11,615 | 13.6% | 25,379 | 13.6% |
|  |  |  | G3/4 | 5763 | 5.7% | 6404 | 7.5% | 12,167 | 6.5% |
|  |  |  | Unspecified | 929 | 0.9% | 882 | 1.0% | 1811 | 1.0% |
|  | IV | Grading | G1 | 609 | 0.6% | 482 | 0.6% | 1091 | 0.6% |
|  |  |  | G2 | 12,429 | 12.4% | 9516 | 11.1% | 21,945 | 11.8% |
|  |  |  | G3/4 | 7086 | 7.1% | 6501 | 7.6% | 13,587 | 7.3% |
|  |  |  | Unspecified | 2814 | 2.8% | 2457 | 2.9% | 5271 | 2.8% |
|  | X | Grading | G1 | 957 | 1.0% | 786 | 0.9% | 1743 | 0.9% |
|  |  |  | G2 | 6598 | 6.6% | 5439 | 6.3% | 12,037 | 6.5% |
|  |  |  | G3/4 | 1956 | 2.0% | 2189 | 2.6% | 4145 | 2.2% |
|  |  |  | Unspecified | 1468 | 1.5% | 1283 | 1.5% | 2751 | 1.5% |
|  | Total | Grading | G1 | 6488 | 6.5% | 5184 | 6.1% | 11,672 | 6.3% |
|  |  |  | G2 | 65,544 | 65.4% | 52,578 | 61.4% | 118,122 | 63.5% |
|  |  |  | G3/4 | 21,029 | 21.0% | 21,666 | 25.3% | 42,695 | 23.0% |
|  |  |  | Unspecified | 7221 | 7.2% | 6257 | 7.3% | 13,478 | 7.2% |

**Supplementary Table 1:** Sex-specific distribution of grading according to UICC stage (overall study cohort; n=185,967). All differences in relative distributions are highly significant with p < 0.001 according to Pearson’s Chi-square test.

| Variable, factor | Category | p | Odds ratio | Lower 95% | Upper 95% |
| --- | --- | --- | --- | --- | --- |
| Year of diagnosis | 2000-04 | <.001 |  |  |  |
|  | 2005-09 | <.001 | .886 | .865 | .908 |
|  | 2010-16 | <.001 | .816 | .797 | .836 |
| Age at diagnosis | 0-49 | <.001 |  |  |  |
|  | 50-59 | <.001 | .803 | .765 | .842 |
|  | 60-69 | <.001 | .693 | .664 | .724 |
|  | 70-79 | <.001 | .874 | .838 | .912 |
|  | 80+ | <.001 | 1.614 | 1.543 | 1.687 |
| Tumor localization | Right colon | <.001 |  |  |  |
|  | Transverse colon | <.001 | .852 | .823 | .882 |
|  | Left colon | <.001 | .729 | .714 | .744 |
|  | Other locations | <.001 | .793 | .760 | .826 |
| Second tumor | No |  |  |  |  |
|  | Yes | <.001 | .724 | .678 | .773 |
| Histolog. type | Adenocarcinoma | <.001 |  |  |  |
|  | Neuroendocr. Ca | <.001 | 1.220 | 1.136 | 1.310 |
|  | Other tumor entities | .291 | 1.032 | .973 | 1.094 |
|  | Other benign lesions | .381 | 1.097 | .892 | 1.349 |
| Grading | G1 | <.001 |  |  |  |
|  | G2 | .779 | .994 | .955 | 1.035 |
|  | G3/4 | <.001 | 1.190 | 1.139 | 1.243 |
|  | Unspecified | .016 | 1.066 | 1.012 | 1.124 |
| UICC stage | I | <.001 |  |  |  |
|  | II | .002 | 1.046 | 1.016 | 1.076 |
|  | III | <.001 | 1.111 | 1.078 | 1.145 |
|  | IV | .839 | 1.003 | .973 | 1.034 |
|  | X | .352 | 1.017 | .981 | 1.054 |
| Constant |  | <.001 | 1.135 |  |  |

**Supplementary Table 2:** Odds ratios from multivariable binary logistic regression for risk of female versus male patients subject to patient demographic, clinical and histopathological characteristics (overall study cohort; n=185,967).

|  | | Sex | | | | | |
| --- | --- | --- | --- | --- | --- | --- | --- |
|  |  | Male | | Female | | Total | |
|  |  | N | % | N | % | N | % |
| Year of diagnosis | 2000-04 | 14822 | 23.7% | 13777 | 25.5% | 28,599 | 24.5% |
|  | 2005-09 | 20910 | 33.4% | 18015 | 33.4% | 38,925 | 33.4% |
|  | 2010-16 | 26834 | 42.9% | 22170 | 41.1% | 49,004 | 42.1% |
| Age at diagnosis | 0-49 | 2810 | 4.5% | 2389 | 4.4% | 5199 | 4.5% |
|  | 50-59 | 7512 | 12.0% | 5569 | 10.3% | 13,081 | 11.2% |
|  | 60-69 | 18603 | 29.7% | 11964 | 22.2% | 30,567 | 26.2% |
|  | 70-79 | 23646 | 37.8% | 19202 | 35.6% | 42,848 | 36.8% |
|  | 80+ | 9995 | 16.0% | 14838 | 27.5% | 24,833 | 21.3% |
| Tumor localization | Right colon | 23222 | 37.1% | 24544 | 45.5% | 47,766 | 41.0% |
|  | Transverse colon | 5453 | 8.7% | 4641 | 8.6% | 10,094 | 8.7% |
|  | Left colon | 31252 | 50.0% | 22536 | 41.8% | 53,788 | 46.2% |
|  | Other locations | 2639 | 4.2% | 2241 | 4.2% | 4880 | 4.2% |
| Tumor localization | Cecum | 8165 | 13.1% | 9808 | 18.2% | 17,973 | 15.4% |
|  | Appendix | 395 | 0.6% | 404 | 0.7% | 799 | 0.7% |
|  | Right colon | 10757 | 17.2% | 11294 | 20.9% | 22,051 | 18.9% |
|  | Right flexure | 3905 | 6.2% | 3038 | 5.6% | 6943 | 6.0% |
|  | Transverse colon | 5453 | 8.7% | 4641 | 8.6% | 10,094 | 8.7% |
|  | Left flexure | 2492 | 4.0% | 1739 | 3.2% | 4231 | 3.6% |
|  | Left colon | 3935 | 6.3% | 2654 | 4.9% | 6589 | 5.7% |
|  | Colon sigmoideum | 24825 | 39.7% | 18143 | 33.6% | 42,968 | 36.9% |
|  | Overlapping loc. | 576 | 0.9% | 463 | 0.9% | 1039 | 0.9% |
|  | Other locations | 2063 | 3.3% | 1778 | 3.3% | 3841 | 3.3% |
| Grading | G1 | 4383 | 7.0% | 3327 | 6.2% | 7710 | 6.6% |
|  | G2 | 44586 | 71.3% | 36392 | 67.4% | 80,978 | 69.5% |
|  | G3/4 | 11271 | 18.0% | 12246 | 22.7% | 23,517 | 20.2% |
|  | Unspecified | 2326 | 3.7% | 1997 | 3.7% | 4323 | 3.7% |
| UICC stage | I | 18381 | 29.4% | 14250 | 26.4% | 32,631 | 28.0% |
|  | II | 24232 | 38.7% | 21262 | 39.4% | 45,494 | 39.0% |
|  | III | 19953 | 31.9% | 18450 | 34.2% | 38,403 | 33.0% |
|  | Total | 62566 | 100.0% | 53962 | 100.0% | 116,528 | 100.0% |

**Supplementary Table 3:** ACO group only (n= 116,528): patient demographic, clinical and histopathological characteristics according to sex. All differences in relative distributions are highly significant with p < 0.001 according to Pearson’s Chi-square test.

|  | | Sex | | Chemotherapy | | | | | | | | |  | | |
| --- | --- | --- | --- | --- | --- | --- | --- | --- | --- | --- | --- | --- | --- | --- | --- |
|  |  |  |  | Yes | | | No | | | Total | | |  | | |
|  |  |  |  | N | | % | N | | % | N | | % | *p** | | |
| Age at diagnosis | 0-49 | | Male | 746 | 80.7% | | 178 | 19.3% | | 924 | 100.0% | | | .824 |  |
|  |  |  | Female | 628 | 80.3% | | 154 | 19.7% | | 782 | 100.0% | | |  |  |
|  | 50-59 | | Male | 1869 | 81.7% | | 419 | 18.3% | | 2288 | 100.0% | | | .513 |  |
|  |  |  | Female | 1404 | 80.9% | | 332 | 19.1% | | 1736 | 100.0% | | |  |  |
|  | 60-69 | | Male | 3841 | 75.1% | | 1274 | 24.9% | | 5115 | 100.0% | | | .014 |  |
|  |  |  | Female | 2760 | 77.4% | | 807 | 22.6% | | 3567 | 100.0% | | |  |  |
|  | 70-79 | | Male | 3938 | 62.9% | | 2327 | 37.1% | | 6265 | 100.0% | | | .418 |  |
|  |  |  | Female | 3393 | 62.1% | | 2068 | 37.9% | | 5461 | 100.0% | | |  |  |
|  | 80+ | | Male | 662 | 25.8% | | 1903 | 74.2% | | 2565 | 100.0% | | | <.001 |  |
|  |  |  | Female | 831 | 19.4% | | 3447 | 80.6% | | 4278 | 100.0% | | |  |  |
|  | Total | | Male | 11,056 | 64.4% | | 6101 | 35.6% | | 17157 | 100.0% | | | <.001 |  |
|  |  |  | Female | 9016 | 57.0% | | 6808 | 43.0% | | 15824 | 100.0% | | |  |  |

**Supplementary Table 4:** Adjuvant chemotherapy according to age group and sex in patients with R0-resected nodal-positive colon carcinoma UICC stage III (n= 40,497). *p-value derived from Pearson’s Chi-square test.
